# Patient preferences of healthcare delivery in irritable bowel syndrome: a focus group study

**DOI:** 10.1186/s12876-021-02030-x

**Published:** 2021-11-23

**Authors:** Gwen M. C. Masclee, Johanna T. W. Snijkers, Marijke Boersma, Ad A. M. Masclee, Daniel Keszthelyi

**Affiliations:** 1 Division of Gastroenterology and Hepatology, Department of Internal Medicine, Maastricht University Medical Center +, PO Box 5800, 6202 AZ Maastricht, The Netherlands; 2 NUTRIM, School of Nutrition and Translational Research in Metabolism, Maastricht University, Maastricht, The Netherlands; 3 Dutch Foundation of Gastroenterology and Hepatology (Maag Lever Darm Stichting), Amersfoort, The Netherlands

**Keywords:** Irritable bowel syndrome, Therapy, Treatment, Focus group, Patient preference

## Abstract

**Background:**

Irritable bowel syndrome (IBS) is a highly prevalent disorder with significant negative impact on quality of life of patients that results in high healthcare use and costs. Improving healthcare outcomes for IBS patients is warranted, however the exact needs of IBS patients with regard to therapy and control of symptoms are unknown.

**Methods:**

Focus group interviews, using a two-stage model, were performed with twenty-three IBS patients meeting Rome III criteria and one mother of a patient, from four different regions from the Netherlands.

**Results:**

Twenty-four participants were included of whom majority were female (n = 21), mean age was 43 years, and mean duration of IBS was 18 years. Five categories of patients’ perspectives were identified: clear communication, a multidisciplinary treatment team, centers of expertise, focus on scientific research and information about IBS that is widely available for patients.

**Conclusions:**

Based on these findings we highlight the need for IBS care givers to take these key items into account in IBS care. These elements aid clinicians, but mostly patients, in coping and management of symptoms and subsequent healthcare outcomes, reducing overall healthcare use and costs.

## Background

Irritable bowel syndrome (IBS) is a disorder of the gut-brain interaction, characterized by recurrent abdominal pain and altered bowel habits. IBS is a prevalent disorder with female predominance [1]. The natural course of IBS includes relapsing and remitting symptoms. As the disorder is chronic, symptoms of IBS patients affect the day-to-day life of patients to a large extent [2–4]. This results not only in increased healthcare utilization, but also in a significant economic burden. Increased health care utilization results in direct costs, e.g. as high as 36% of total gastrointestinal related healthcare costs in the Netherlands[5], however, also in indirect costs in terms of loss of work productivity and absenteeism [6, 7]. Current treatment relies on explanation of the diagnosis, reassurance, life style advice, and pharmacological and non-pharmacological treatment regimens [8]. International guidelines up to now, describe clinical and technical details of treatment strategies, but lack understanding of therapy implications from a patient’s perspective. [9–11] A key element for IBS care includes a strong therapeutic physician–patient relationship that allows individualized advice for each patient. IBS is a disorder in which there is no cure, requiring patients to develop coping skills in order to deal with symptoms on a daily basis. Reducing the need for professional assistance, for instance by self-management plans, may increase cost-effectiveness and allow clinicians to focus more on patients with complex problems [12, 13]. Up to now, only a limited number of studies focused on patients’ preferences for treatments and mode of information delivery in IBS patients [14–19]. Although many IBS patients are treated each day by their healthcare provider and are given advice for different symptoms, physicians may not be fully aware of patients’ specific needs and wishes with respect to treatment choices and strategies. In order to tailor treatment to individualized needs in IBS patients, qualitative research is essential. The limited number of previous qualitative studies in IBS however have not addressed patients’ preferences in general in current IBS care. In the current focus group study our aim was to assess the needs of IBS patients with regard to preferences in healthcare delivery. We hypothesized that clear communication is one of the essential elements for IBS patients’ needs.

## Methods

### Recruitment of participants

Participants between the age of 18 and 75 years from four different regions of the Netherlands to ensure geographical diversity (Amsterdam, Amersfoort, Maastricht and Zwolle) were included in the study. Participants were recruited between December 2018 and January 2019 in several ways: via participating in previous research [20], and Maastricht IBS Cohort NCT00775060, the outpatient department of Gastroenterology and Hepatology, via general practitioners, via a patient peer group website and via a specialized recruitment agency. Participants were diagnosed with IBS in the past by a physician according to Rome III criteria. In addition, the mother of one of the participants attended the focus group interviews. IBS diagnoses were confirmed by a questionnaire on paper, which participants had to complete before the focus group interview. During completion of the questionnaire, a physician (JTWS) was present for questions, and confirmed IBS diagnosis. Participants with organic diseases possibly explaining the gastrointestinal symptoms, such as celiac disease or inflammatory bowel disease, as diagnosed by a physician, were not eligible for inclusion. Furthermore, participants with prior abdominal surgery (except appendectomy, laparoscopic cholecystectomy, and hysterectomy) were excluded. Participants needed to understand written Dutch and speak the Dutch language, as focus groups were conducted in Dutch. All subjects gave permission and informed consent for audio recording prior to participation. Informed consent was obtained from all subjects or, if subjects are under 18, from a parent and/or legal guardian. According to Dutch legal regulations, protocols for focus group interviews are not subject to formal review by the ethics committee as determined by the ‘Wet medisch-wetenschappelijk onderzoek met mensen (WMO)’. All study procedures were conducted in accordance with the Declaration of Helsinki and all other relevant guidelines and regulations. Standards for reporting qualitative research (SRQR) were followed [21].

### Focus group interviews

The method of focus group interviews as used in the current study, has been described previously [20, 22]. An initial draft with essential elements of IBS care was constructed by several national experts in the field of neurogastroenterology and motility. Meetings took 145 min and were planned one by one, including five to seven participants each. Group discussions continued until subjects felt all items were discussed until satisfaction and until saturation (i.e. until no longer new items or information was addressed). Focus groups were guided by an independent moderator and at least one assistant-moderator (JTWS). Interviews were performed according to a predefined, two-stage framework [15]. The first stage included an open discussion in which participants were questioned about their previous experiences and aspects of their IBS care, which they considered to be essential to improve care for other and future IBS patients (see specific questions in Table 1). In the second stage, specific items and suggestions as made by the input of the experts were open for discussion in the group interviews. To stimulate an open and active discussion, participants were asked to choose pictures that represented their feelings and expectations about expert centers for IBS care. A two-stage framework was chosen to prevent introducing subject items and introducing bias. The questionnaire and item list used in the interview is shown in Table 1.

**Table 1:** Questionnaire and item list used for focus group interviews

*Experiences with current IBS care*
Describe your experience with the care for IBS patients
In particular for care around the time of your IBS diagnosis, how did you experience care (1) before; (2) during and (3) after IBS diagnosis; which aspects were taken good care of and which aspects could deserve improvement?
When looking back at the time period before your IBS diagnosis, which were your biggest frustrations, which elements did you miss the most?
What are your feelings about healthcare givers during the time period of your IBS diagnosis? From whom did you get advices, were these helpful and why?
*Expectations for future IBS care*
Describe your ideal care for future IBS patients
How do you feel about centers of expertise for IBS care?
Which standards of care are needed for these IBS centers of expertise?
Which type of healthcare givers would you like to be involved in IBS centers of expertise?
What should these IBS centers of expertise look like?
Which items are essential for such centers; what should be taken into account?
*Specific items for open discussions*
Diagnostic elements that should be performed to confirm diagnosis of IBS
Collaboration between general practitioner and medical specialist
Collaboration between healthcare givers: physicians, nurses, dieticians, psychotherapists, others
Different treatment options for IBS
Location of IBS care and IBS centers of expertise
Availability and findability of IBS care and IBS centers of expertise
Type of information available (online, pamphlet) and modus of intervention (face-to-face contact, web-based, telephone based)
Information for patients

### Analysis of focus group data

Focus group interviews were recorded by audio and additionally, attending investigators made notes. Audio recordings were transcribed verbatim after the meetings and subsequently summarized in a spreadsheet database. Subsequently, this spreadsheet was used to evaluate and combine results from all focus group meetings in order to present in this paper. A pre-specified framework of items was used to group results from the second stage of group interviews into several general items in order to facilitate implementation. Items raised by the first stage of group interviews could be added to this list. Demographic and clinical information was obtained through questionnaires that were filled out by the participants before the focus group discussion began. Descriptives of demographics were analyzed by SPSS.

## Results

### Study participants

**Fig. 1:**
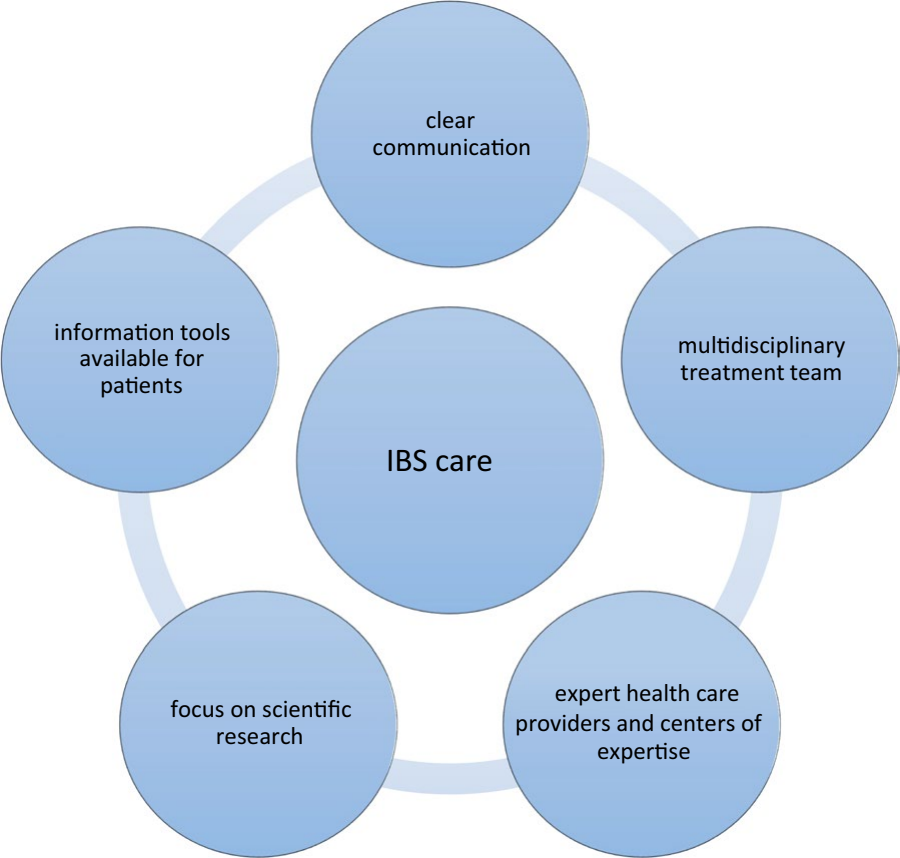
Suggested key elements for IBS care as derived from focus interview group discussions. Multidisciplinary treatment team consisting of a dedicated gastroenterologist, dietician, a nurse physician, and when possible also a hypnotherapist, psychologist and the general practitioner

In total 24 participants were included, including 23 IBS patients. One participant included in the focus group interviews was the mother of a patient diagnosed with IBS according to Rome III criteria. Majority of included participants were female (n = 21), they were middle-aged (mean age 43 years, standard deviation 13 years), mean IBS symptom severity score (IBS-SSS) was 278 (normal range 0–500) and patients suffered from IBS for mean duration of 18 years (standard deviation 13 years). Results from focus group interviews were clustered in the following themes: (1) communication, (2) multidisciplinary treatment team, (3) expert health care providers and centers of expertise, (4) scientific research, and (5) information tools for patients (Fig. 1).

### Communication

In response to the question about experiences with current IBS care, participants repeatedly stressed the need for clear and open communication. In previous experiences participants felt that health care givers did not communicate in an open manner. The greatest disappointment reported by participants is the fact that there is not a holy grail as an ultimate treatment solution. However, they felt that open communication and being clear on this issue is essential for further understanding of the disease and taking control of their symptoms (“*I was told that I do not have inflammatory bowel disease, nor celiac disease, nor any other disease, so it should be irritable bowel syndrome. I felt not taken seriously at all, as the physician only mentioned what I do not have as a disease*”). Besides, not the lack of a solution for their IBS complaints was felt as a major frustration, but particularly the lack of understanding by their health care provider. Open communication, with acknowledgement of complaints and the impact of symptoms on patients’ lives, is therefore essential. Taking the time to hear and listen to the patients’ story was suggested as key item in the treatment-relationship with the health care provider (“I would like the physician to notice that I am not just a patient, I also have a life with work and a family, which is heavily affected by my IBS, but I’m more than just a number or a patient”). It was not explicitly mentioned by participants that the health care provider taking the time to explain IBS symptoms and possible therapeutic strategies should be a physician. In fact, participants felt that a nurse practitioner probably would have more time available to guide patients in more depth and detail than a physician. They suggested that nurses should be included in the multidisciplinary team (see next item).

### Multidisciplinary treatment team

Participants emphasized the need for a multidisciplinary dedicated treatment team, including a physician, a dietician, a nurse practitioner (e.g. nurse with specialized training in gastroenterology and IBS that can work in collaboration with physicians or work independently) and perhaps even other health care providers (hypnotherapist or psychologist). Patients reported that food and alterations in diet can have a huge improvement on their symptoms and quality of life. However, patients felt that adjusting their diet by themselves is too hard and too complex and guidance by a dedicated dietician is wanted. Some participants had previous experiences with dieticians whose suggestions were too general, whereas other were satisfied with an individualized specific diet plan (“I went to a dietician to help me adjust my diet, I left after one consult and felt I had to figure out myself which food items were increasing my complaints. I had hoped to receive guidance and help from an experienced dietician to ensure a complete and enriched diet when restricting certain food categories”). Furthermore, physicians, but also other health care providers, with up-to-date information on most recent developments on diagnosis and treatment options should be included in the treatment team, as patients reported that some general practitioners (GPs) did not have enough experience with complex symptoms of IBS. Patients think that primary IBS care can be provided by the general practitioner, but high-standard knowledge of some GPs on IBS is currently lacking. (“When I visited the general practitioner for advice on my symptoms, I had to come up with suggestions for therapy by myself”). GPs are preferably included in the multidisciplinary treatment team to increase knowledge about IBS among GPs and to assist in the treatment and guidance of IBS patients. Participants mentioned that approaching your general practitioner is considered easier and quicker than approaching a medical specialist in the hospital. They also suggested that educational training of GPs by IBS expert physicians should be offered. Patients confirmed the preference for a treatment approach with a step-by-step strategy. A clear and well-defined stepwise treatment plan is considered to improve their experiences with the treatment process.

### Expert health care providers and centers of expertise

All participants responded enthusiastic to the suggestion of IBS centers of expertise. They feel the need to have a center where IBS care is up-to-date with high standards, where all expertise from different sources is combined, communication between different health care providers is clear and open, and there is a focus on scientific research. Participants hope that these centers of expertise can provide guidance to all IBS patients, but understand that the majority of IBS patients can be treated and guided successfully by local health care providers such as GPs. It was felt that understanding and acknowledgement of the disease by experts in IBS centers of expertise may aid the patients with IBS in whom the impact on daily life is largest, e.g. those who cannot work or continue day-to-day life due to IBS symptoms or related anxiety and stress disorders (“Only health care providers that truly understand IBS should work at a center of expertise, so the health care provider can understand what I’m going through every day”). Suggested health care providers that should be involved in an expert center include a gastroenterologist, dietician, nurse, and a psychologist, preferentially in close cooperation with the general practitioner. Combining expertise of these health care providers located in the same clinic should enable patients to visit the several health care providers consecutively during the same clinic session and without delays. This also solves the issue raised by participants of finding the right health care providers by themselves. Also, participants were asked to choose pictures that represented their expectations about the expert centers and these included pictures on collaboration, learning experiences, giving guidance and tools for IBS care.

### Scientific research

Participants feel that more scientific research is needed in IBS in order to gain insight into the pathophysiology and treatment options for IBS. (“There should be more research on IBS and treatment, as currently, I’m figuring out myself other and new treatment options”). Also, participants find it difficult to retrieve information on IBS. They hope that more research may aid in finding a solution to the disorder. They show great willingness to participate in scientific research. Currently, participants felt that communication about the scientific research performed by the physicians may be improved. This should be targeted at IBS patients but also to (general) physicians to provide them with the most recent developments in IBS care and research.

### Information tools for patients

Participants mentioned they feel abandoned in gathering information about pathophysiology of IBS and treatment of IBS symptoms; e.g. medical specialists do not have time for day-to-day advice whereas the general practitioner may lack specific information on the newest treatment insights. When asked about information available online, they felt correct information is scattered across the internet and they did not know where to find correct information (“Is there a uniform website for IBS patients? I did not know that at all, I usually browse online and end up with stories of other patients as source of information”). There is lack of a uniform information tool or website with scientifically based information on IBS and treatment options with IBS patients as target audience. Development of a widespread available tool by an expert center or expert panel with up-to-date information on IBS including information on symptoms, pathophysiology, and treatment options was suggested by participants. This allows gathering of information with a low threshold. This information tool should—according to the participants—be promoted by physicians and a peer group website in order to increase publicity.

## Discussion

In the current study we assessed the needs of IBS patients for improvement of IBS care in the Netherlands. We found that communication, information, IBS expertise, scientific research and a multidisciplinary team are paramount to patients’ preferences. When incorporating these items in clinical practice this should enable patients to better control their symptoms and improve coping strategies. Patients included in this study have been suffering from IBS for a mean duration of eighteen years representing a long time span of experience with several therapies, advanced self-care for symptom management and control in daily life. Results of this study are important for healthcare givers and IBS patients in order to improve IBS healthcare outcomes.

Research into effective therapeutic strategies for IBS has primarily focused on establishing the clinical efficacy of specific treatment entities, such as dietary interventions or pharmacotherapy. However, the context in which these treatments are provided might be equally important and has hitherto not been sufficiently examined. In recent years, a few qualitative studies on patients’ preferences for specific treatments of IBS have been published [14–19], however have not focused on patient preferences in general in healthcare delivery. Currently, huge efforts are made for management of IBS symptoms and difficulties in daily life to be based on patient-centered care allowing patients to be the expert and being competent to manage their illness on a day-to-day level. Such strategies include education, cognitive and behavioral approaches to increase knowledge and improve symptom management [16, 19] that can be augmented by dietary adjustments, pharmacotherapy, or more complex psychological interventions. Symptom management can often involve multiple attempts involving different therapeutic modalities before arriving at a sufficiently satisfying clinical response. Apart from this, it is important to consider in which modality therapy or information is given. Patients’ experiences and preferences of source and type of information varies from detailed book advice to physical support groups to widespread available internet pages [12, 18, 23]. An individual choice of therapy is important to improve therapy adherence and success[24], and providing relevant and clear information for patients is essential for this choice. However, it remains largely unknown which factors in IBS care are important for patients. In recent years qualitative research has gained in popularity as it allows—in contrast to quantitative research—assessing illness experience such as severity of IBS or quality of life [14, 15]. Particularly since efficacy in terms of number needed to treat for all treatment options for IBS is similar, [25] it is of much more importance that we currently understand how we should successfully approach, treat and manage individual patients. While other qualitative studies in IBS patients focused on the patient experience of living with IBS, on specific treatment modalities, or on healthcare in general, the current study shows that the mode of delivery of treatment information is important and is currently not addressed in IBS care. Focus group interviews provide a nuanced perspective of patients’ experiences of illness that cannot be captured in questionnaires or surveys. As previously shown in a focus group study, IBS patients have varying opinions on different sources of information that may be available [12, 26]. In addition to previous studies [14, 15, 19], we found that communication and understanding of the patient perspective is important for patients’ needs in IBS care. Importantly, it highlights the needs for diagnosing IBS using the positive diagnostic criteria, as formulated in the Rome IV consensus [27], rather than considering IBS as a diagnosis of exclusion. This way, the patient feels that the complaints are not being delegitimized and are taken seriously—as was also expressed by patients in the current study. In addition, our study reveals that when considering medical support for IBS, involvement of a multidisciplinary team is important. Although guidelines suggest a variety of management options, there are no recommendations on combining medical expertise in one specific or center of expertise [10, 25]. Based on our findings, patients feel that the knowledge of dedicated IBS health care providers, communication between health care providers in centers of expertise and availability of a variety of health care providers (physician, nurse, dietician and psychologist, preferably at the same location) will improve healthcare and outcomes for IBS patients. Therefore, these items are important to take into account in future IBS care. Indeed, a recent study from Australia showed that integrated multidisciplinary clinical care was superior to gastroenterologist-only care in relation to symptoms, psychological state, quality of life, and cost of care for the treatment of functional gastrointestinal disorders, including IBS [28]. One may speculate that undergoing treatment in a center of expertise may help patients feel they have been taken seriously, which is crucial to achieve treatment success. Centers of expertise can also attract significant number of patients for the necessary clinical volume to support a registered dietitian or behavioral therapist as a part of the integrated care model. Another important issue raised in the current study is the person as source of support for patients. We noticed that it is not as important for patients who the person is explaining symptoms or guiding in management, e.g. physician, nurse or dietician, as long as they have expertise with IBS and up-to-date knowledge.

Treatments can effectively be supplemented by additional information provided to the patient in writing. In the absence of such being supplied from the healthcare provider, as is often the case, patients frequently join online forum groups for this kind of information, in addition to emotional support. Unfortunately, the information provided is not necessarily scientifically proven [29]. By providing information from approved sources to support patients, and providing an overview of therapeutic options, patients show improved adherence to a chosen treatment. Subsequently, control of their IBS symptoms and quality of life improves while healthcare utilization and healthcare costs are reduced. If we are able to counsel our IBS patients with an (interactive) tool with widespread availability and easy to understand, this should decrease patients’ use of healthcare [7] and more importantly, associated health care costs [6].

Strengths of the current study include the use of focus group interviews, which is a standardized and validated method to assess patients’ perspectives [15]. To the authors’ knowledge, this is the first study exploring patients’ preferences for IBS care in general rather than for specific therapies. Also, participants were suffering from IBS for many years and thus have had a long time of experience of trying a range of treatment and coping strategies. As for patient preferences, the second part of the interviews contained pre-selected items which participants were requested to comment on and therefore a bias could have been introduced, but this was intentional. Other limitations of the current study include the voluntary participation in focus groups, thereby potentially limiting the representation of the whole IBS population. We have mitigated against this by recruiting patients from different sources (e.g. hospitals, general practitioners, an unbiased recruitment agency and via peer group websites). Nevertheless, the patients included might not be representative of those individuals experiencing IBS-symptoms but not having received a formal diagnosis. The majority of participants in our study were female (91%), which is common for functional GI disorders [30]. However, previous studies have shown that the impact of IBS symptoms on the quality of life is larger in women than in men. Particularly, women report more fatigue, more feelings of anxiety and depression and less self-control [31]. In female patients, social gain of treatment on symptoms and quality of life is the largest, therefore we feel that the selected group of participants preferentially represents the group emphasizing the need of IBS patients the best. Also, those who participated in the groups attended mainly because they were seeking outside help or were hoping to find a new cure. We kept the number of participants in the focus group meeting by design small (i.e. five to seven) in order to stimulate all participants to participate actively in the discussions, as done previously [15, 20, 22]. As this study was conducted in the Netherlands, some aspects might be related to specific local aspects of healthcare services, although we believe that our findings can be generalized for IBS-related healthcare delivery. Although participants were diagnosed based on Rome III criteria, IBS patients still represent an inhomogeneous group of patients, with a pathophysiology that is still not completely understood, although current consensus considers IBS as being a disorder of the gut-brain interaction. Regardless of the exact underlying pathophysiology, we believe stressing a ‘positive diagnosis of IBS’ is key for symptom management. However, one should realize that in clinical practice there is still uncertainty about disorders or diseases that we may unravel in the future, that may be associated with symptoms of IBS.

## Conclusions

In conclusion, we show that essential items in IBS care according to patients’ preferences include communication, information, medical expertise, a multidisciplinary treatment team and scientific research. Findings of the current study highlight the need for better understanding of patients’ preferences with regards to how treatment is delivered and identifies key areas of improvement. The results from this focus group study will aid not only patients but also physicians in optimizing and enhancing care of IBS patients as in terms of healthcare outcomes and quality of life.

## Data Availability

The datasets used and/or analysed during the current study available from the corresponding author on reasonable request.

## Abbreviations

IBS: Irritable bowel syndrome
GP: General practitioner
